# Mechano-chemical Interactions in Cardiac Sarcomere Contraction: A Computational Modeling Study

**DOI:** 10.1371/journal.pcbi.1005126

**Published:** 2016-10-07

**Authors:** Lauren J. Dupuis, Joost Lumens, Theo Arts, Tammo Delhaas

**Affiliations:** 1 Department of Biomedical Engineering, Cardiovascular Research Institute Maastricht (CARIM), Maastricht University, Maastricht, The Netherlands; 2 Université de Bordeaux, LIRYC L'Institut de Rythmologie et Modélisation Cardiaque, Bordeaux, France; University of California San Diego, UNITED STATES

## Abstract

We developed a model of cardiac sarcomere contraction to study the calcium-tension relationship in cardiac muscle. Calcium mediates cardiac contraction through its interactions with troponin (*Tn*) and subsequently tropomyosin molecules. Experimental studies have shown that a slight increase in intracellular calcium concentration leads to a rapid increase in sarcomeric tension. Though it is widely accepted that the rapid increase is not possible without the concept of cooperativity, the mechanism is debated. We use the hypothesis that there exists a base level of cooperativity intrinsic to the thin filament that is boosted by mechanical tension, i.e. a high level of mechanical tension in the thin filament impedes the unbinding of calcium from *Tn*. To test these hypotheses, we developed a computational model in which a set of three parameters and inputs of calcium concentration and sarcomere length result in output tension. Tension as simulated appeared in good agreement with experimentally measured tension. Our results support the hypothesis that high tension in the thin filament impedes *Tn* deactivation by increasing the energy required to detach calcium from the *Tn*. Given this hypothesis, the model predicted that the areas with highest tension, i.e. closest to the Z-disk end of the single overlap region, show the largest concentration of active *Tn*’s.

## Author Summary

Cardiac contraction is the culmination of multiple subcellular processes beginning with calcium induced activation of the contractile machinery. Interestingly, small increases in intracellular calcium concentrations lead to disproportionately large increases in tension development within the cardiac muscle, a phenomenon known as ‘cooperative activation’. Although the concept of cooperative activation is widely accepted, the mechanism is highly debated. Many complex computational models have been developed in an attempt to understand the underlying mechanisms. However, no single mechanism has been able to properly account for the range of experimental data. We propose that the interaction between the mechanics and chemistry in the contractile unit is an essential component of the cooperative activation. We have developed a simple computational model composed of only five parameters and a single ordinary differential equation that describes the cooperative nature of myocardial contraction through mechanochemical interaction. This model can reproduce experimental data relating to cooperative activation and provides a promising tool for future research.

## Introduction

It has been widely known since the 1883 work of Ringer [1] that calcium ions (*Ca*^*2+*^) regulate muscle contraction. Although the mechanism was unknown at the time, Ringer found that rat hearts did not contract forcefully unless *Ca*^*2+*^ was added to the solution perfusing the muscle. Since Ringer’s discovery the relationship between *Ca*^*2+*^ concentration ([*Ca*^*2+*^]) and tension in muscle has been widely studied. Experiments have shown a steep rise in steady state tension with relatively little increase in intracellular [*Ca*^*2+*^]. The desire to understand the steepness of the [*Ca*^*2+*^]—tension relationship has led to the hypothesis that cardiac muscle contraction is highly cooperative [2, 3]. Cooperativity implies that a single event encourages subsequent similar events. In the case of muscle contraction, a single *Ca*^*2+*^ binding invoking the generation of tension might enhance additional *Ca*^*2+*^ bindings to generate more tension [4]. Various hypotheses about the mechanism of cooperativity in cardiac muscle have been proposed.

**Fig 1:**
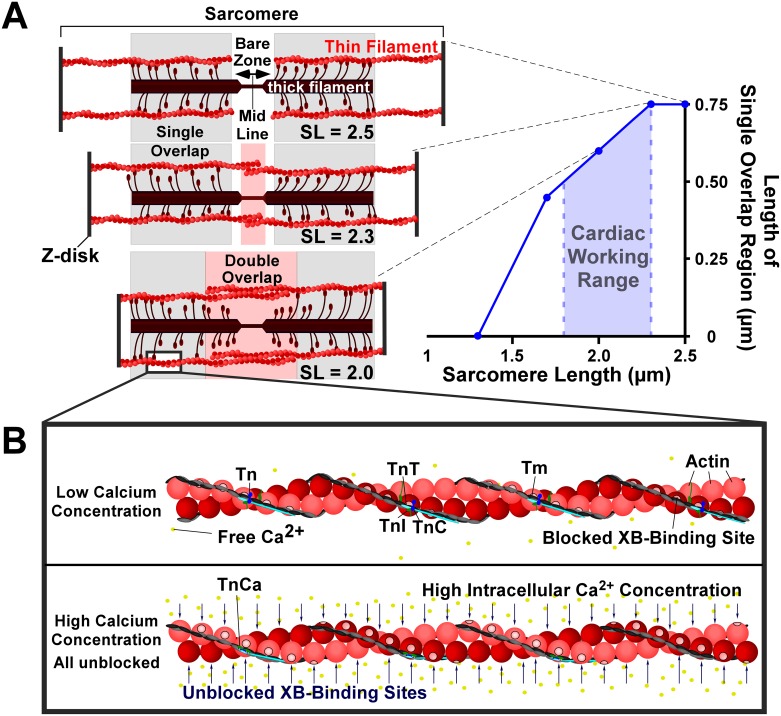
Schematic representation of sarcomere. (A). Sarcomeres are composed of thick and thin filaments that interact to generate force. Sarcomere length (*SL*) determines the length of single overlap region where cross-bridges (*XB*) can form and generate force. (B) At rest, the binding sites on the spherical actin are blocked by a strand of tropomyosin (*Tm*). *Ca* binding to *Tn* (*TnCa*) activates a troponin complex (*Tn*) resulting in the lateral movement of the *Tm* strand that unblocks the *XB*-binding sites. The *Tn* is composed of three subunits, troponin C (*TnC*), troponin I (*TnI*), and troponin T (*TnT*).

Before discussing cooperativity, we explain the structures and molecular mechanisms involved in cardiac contraction. The cardiac muscle is striated, meaning that it is composed of a repeating organization of contractile units known as sarcomeres (Fig 1A). Within the sarcomeres thick and thin filaments can interact with one another to generate tension [5, 6].

The sarcomere is bounded by two opposing Z-disks with the thin filaments protruding from each (Fig 1A). A thin filament contains a double helical arrangement of spherical actin monomers (Fig 1B), each with a myosin binding site on its surface facing the outside of the helix [7]. A thin filament is surrounded by thick filaments, each having myosin heads protruding every 14.3 nm that can bind to actin to form a cross-bridge (*XB*) that can generate force [8]. The thick and thin filaments range in length from ~1.5 to ~1.7 μm and ~1.1 to 1.3 μm, respectively [9]. The distance between Z-disks is equal to the sarcomere length (*SL*). Opposing thin filaments can mutually overlap in the so-called double overlap region (Fig 1A). The single overlap region is the section of the thick filament outside of the double overlap region that overlaps with the thin filament. We assume that *XB*’s can form solely in the single overlap region [10]. As *SL* increases up to about 2.3 μm, the length of the double overlap region decreases, thereby lengthening the single overlap region, thus providing more opportunities for *XB* formation and subsequently higher force generation (Fig 1A).

**Fig 2:**
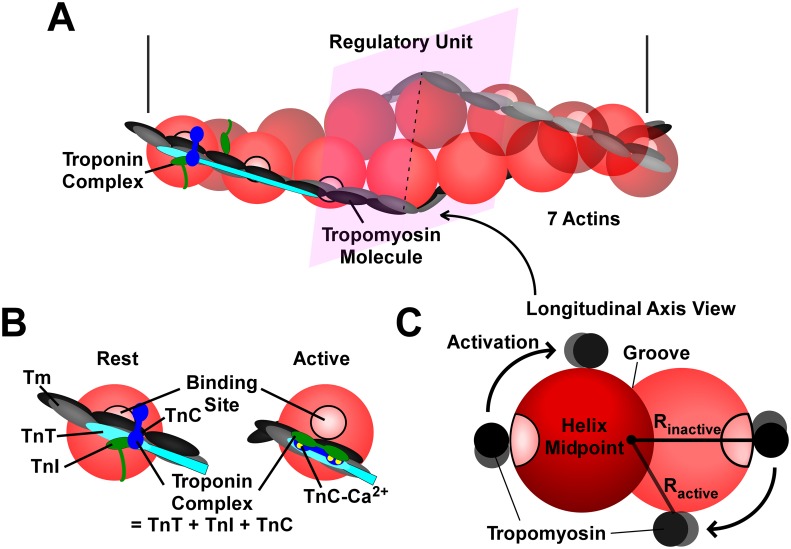
Schematic representation of *Tn* activation. (A) A *RU* is a functional unit of the sarcomere that is considered to work together. It is composed of 7 actin monomers, one tropomyosin (*Tm*) molecule, and one troponin complex (*Tn*). At rest, the C-terminal section of *TnI* extends over the actin monomer on the adjacent strand reaching to the *Tm* molecule, hindering its movement [17]. That hindrance is removed upon the conformational changes resulting from *Ca*^*2+*^ binding to that *Tn*. Note: *Tn*’s on adjacent actin strands are located in-register with one another, but this is not displayed in the figure. (B) A conformational change in the *Tn* caused by the binding of calcium to *TnC* effectively removes the *Tm* from the blocking position. (C) The thin filament in (A) is viewed from the longitudinal axis. The movement of *Tm* from the position blocking the *XB*-binding sites toward the groove reduces the radius (R) from the center of the actin double helix.

*Ca*^*2+*^ activates the thin filament, allowing *XB*’s to form and force to develop (Fig 1B). Seven actin monomers, one tropomyosin (*Tm*) molecule, and one troponin complex (*Tn*) form a repeating structure called a regulatory unit (*RU*) along the thin filament (Fig 2A) [11]. *Tm* molecules are double helical molecules that follow the same helical path as the actin monomers, overlapping one another head to tail under the *Tn*. At rest, the *Tm* molecule wraps around the actin monomers in a position that blocks the myosin binding sites, thereby hindering the formation of *XB*’s and thus contraction. Each *Tn* is composed of three subunits, the inhibitory subunit (*TnI*), the *Tm* binding subunit (TnT) that attaches the *Tn* to *Tm*, and the *Ca*^*2+*^ binding subunit (*TnC*) [12](Fig 2B). *TnI* binds to actin monomers at rest and essentially anchors the *Tm* molecule in place, blocking the binding sites on actin [13]. When the cell depolarizes, *Ca*^*2+*^ enters and may bind to *TnC* opening a hydrophobic patch on the N-terminus of *TnC*. The COOH terminus of *TnI* exhibits a strong affinity for the NH_2_ terminus opened on *TnC* and binds strongly to it, and the inhibitory peptide of *TnI* releases actin[14]. The *Tm* strand moves laterally, thus freeing nearby *XB* binding sites on the thin filament (Fig 2C). We refer to the latter movement of *Tm* as the activation of the *Tn* (Fig 2B). It has been shown that, along with *Ca*^*2+*^ binding to *TnC*, the formation of a strong *XB* is necessary to fully unblock the binding sites on actin [15]. During relaxation the [*Ca*^*2+*^] in the cell decreases, *TnC* releases the bound *Ca*^*2+*^ ions, and *TnI* rebinds to actin moving the *Tm* back over the *XB*-binding sites preventing further force generation [16].

Commonly the relation between intracellular [*Ca*^*2+*^] and muscle tension is described by the Hill-relation, having zero active tension at low [*Ca*^*2+*^] and approaching a level of saturation at high [*Ca*^*2+*^]. If a single *Ca*^*2+*^ ion would bind to a single *Tn* complex without cooperativity, for a physiological muscle contraction, the required increase in [*Ca*^*2+*^] would be unphysiologically large. By comparing the relatively small, physiological change in [*Ca*^*2+*^] with the resulting large change in muscle tension, *Ca*^*2+*^ binding to *Tn* requires an increase of cooperativity by the so-called Hill coefficient *n*. In experiments by Dobesh et al. [18] it is found n≈7.

On the basis of conventional chemical principles, such a high level of cooperativity requires full cooperation between a number of binding sites at least equal to the Hill coefficient. In cardiac muscle, each *Tn* contains three binding sites for *Ca*^*2+*^. Each of the two intertwined actin strands in the thin filament contains a *Tn* after every sequence of seven actin monomers. The *Tn*’s of both actin strands located at equal lengths form repetitive pairs of *Tn*’s, or in-register *Tn*’s. Galińska-Rakoczy et al. showed that the C-terminal section of *TnI* extends over the actin monomer on the adjacent strand reaching to the *Tm* molecule [17]. Their results suggest that upon *Ca*^*2+*^ binding the *Tn* may influence the position of the *Tm* molecule on the surface of the adjacent actin strand as well. Because of this interaction, we consider some degree of cooperativity of *Ca*^*2+*^ binding to in-register *Tn*’s. Each *Tn* complex has 3 binding sites for *Ca*^*2+*^, with one of them having a substantially lower affinity for *Ca*^2+^ than the others [19]. So, potentially, each pair of in-register *Tn* complexes contains 6 binding sites, limiting the intrinsic Hill coefficient to a maximum value of 6 if each of the binding sites exhibits an equal degree of cooperativity. The term ‘intrinsic’ is used to indicate that the binding is considered purely chemical, excluding effects of mechanical tension. In experiments by Sun et al. [20], in the absence of muscle tension, *Ca*^*2+*^ was bound to *Tn* with a Hill coefficient of 3, indicating that the intrinsic cooperativity was about half of the abovementioned maximum. Even in the case of full cooperativity between in-register *Tn*’s, i.e. with an intrinsic cooperativity coefficient equaling 6, one more binding site on a *Tn* in the next *RU* would be necessary to reach a cooperativity represented by the Hill coefficient of 7 in experiments of Dobesh et al. [18]. However, *Tn*’s are separated along the thin filament *Tn*’s by about 35 nm, a distance too large for conventional mechanisms of chemical cooperativity.

In many current sarcomere models, the trend is to represent cooperativity along the thin filament as the interaction between neighboring *XB*’s and *RU*’s. Razumova and colleagues [21] developed a stiffness-distortion model of the *XB* and tested three different models of nearest neighbor cooperative interactions (*RU*-*RU*, *XB*-*XB*, *XB*-*RU*). They found that while none of these interactions were able to completely account for the cooperative force development observed in experiments, each mechanism had a large impact on the [*Ca*^*2+*^]-tension relationship. *XB*-*XB* interactions had the highest impact on peak force, *RU*-*RU* interactions had the greatest impact on the steepness of the [*Ca*^*2+*^]-tension relationship, and *XB*-*RU* interactions had the greatest impact on *Ca*^*2+*^ sensitivity. The model as developed by Rice and colleagues [22] was based on the model of Razumova and colleagues [21] including assumed nearest neighbor cooperativity. Campbell and colleagues [23] have developed a model of cooperativity in which adjacent *Tm* molecules overlap one another at the *Tn*, thereby encouraging *RU* activation when the nearest neighbor *RU* is activated. It is not clear yet on what physical principles such *RU*-*RU*, *RU*-*XB* and *XB*-*XB* interactions would work.

It has further been proposed that at longer *SL*’s the constant volume of the myofiber lattice causes the filaments to squeeze closer together, possibly encouraging binding events by moving a myosin head within reach of a binding site on the thin filament [24, 25]. Similarly, Daniel et al. propose that the cooperative realignment of binding sites occurs when the thin filament is strained by up to 2 nm [26], a lateral movement that could possibly move a binding site within reach of a myosin head. Still others believe that the end-to-end interactions of the *Tm*’s along the thin filament are responsible for the cooperative activation [27].

In the present study we introduce a mechanism based on the mechanochemical interaction of *Ca*^*2+*^ binding to *Tn*. Binding of *Ca*^*2+*^ to *Tn* results in conformational changes in the *Tn* complex. We postulate that mechanical stretch of the thin filament facilitates the change in conformation by reducing the energetic increment required for binding of *Ca*^*2+*^ to *Tn*. Thus, mechanical tension in the thin filament will shift the equilibrium of *Ca*^*2+*^ binding to *Tn* towards the bound state. The latter shift in equilibrium is quantified by the use of the general physical principle that the ratio of prevalence of two states depends on the difference in energy between the two states. We think that such a mechanism shows properties of cooperativity even though it differs from a pure chemical mechanism.

On the basis of the abovementioned mechanochemical principle, we designed a computational model of cardiac sarcomere mechanics that we call the MechChem model. The MechChem model is intended to predict the tension in a thin filament as a function of [*Ca*^*2+*^] and sarcomere length. We investigated whether the mechanochemical mechanism can boost cooperativity from the intrinsic level with a Hill coefficient of 3 described by Sun et al. [20] up to the level of 7 shown by Dobesh et al. [18] in skinned muscle preparations. The energy to bind *Ca*^*2+*^ to *Tn* is assumed to decrease linearly with thin filament tension. Currently we focus on the static behavior, implying that thin filament tension was calculated while maintaining constant sarcomere length and [*Ca*^*2+*^]. Under static conditions we considered the chemical reactions involved to be in equilibrium.

In investigating the static behavior of cardiac muscle contraction, muscle tension as simulated with the MechChem model is compared with simulations based on conventional chemical binding of *Ca*^*2+*^ to *Tn* with a high degree of cooperativity, resulting in the typical S-shaped Hill curve. Experimental results to compare with were obtained from skinned muscle preparations, subject to various static levels of [*Ca*^*2+*^] and sarcomere length as reported by Dobesh et al [18]. When fitting both the MechChem model and Hill-type model to experimental findings, a [*Ca*^*2+*^]-tension relation is found for each sarcomere length, requiring the estimation of 3 parameters. Furthermore, it is investigated which parameters values of the MechChem model could remain while changing sarcomere length.

## Methods

### Model design

Formation of *XB*’s is initiated by the binding of *Ca*^*2+*^ to *TnC* which we will refer to as the binding of *Ca*^*2+*^ to an in-register pair of *Tn*, referred to in the equations as *Tn*_*2*_. The related chemical equilibrium reaction is represented by

$$Tn_{2}+n\;Ca^{2+}⇆Tn_{2}Ca_{n}$$ (1)

**Fig 3:**
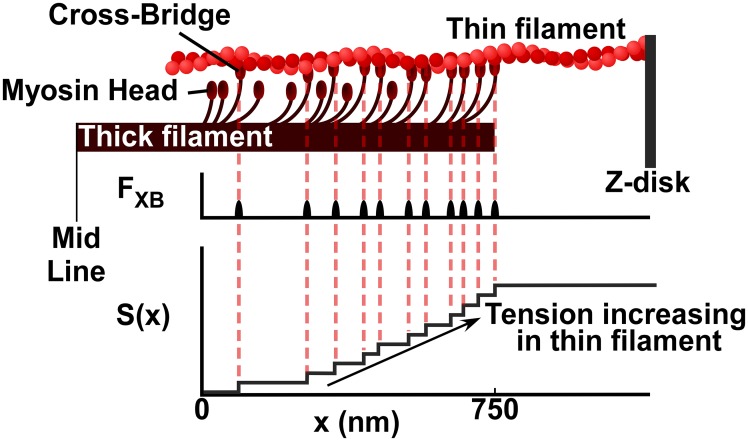
Build-up of tension in the thin filament. Each cross-bridge (*XB*) generates an individual force *F*_*XB*_. Tension *S*(*x*) at location *x* is the sum of each individual *XB* force from the start of the single overlap region closest to the mid line (*x* = 0) to the position *x*. So, tension along the thin filament increases with each *XB* exerting force.

Parameter *n* indicates the coefficient related to the intrinsic cooperativity of *Ca*^*2+*^ binding to *Tn*_*2*_. When *Ca*^*2+*^ binds to *Tn*, *Tm* moves away from the position in which it blocks the *XB*-binding sites on the actin monomers. We assume that the unblocking of *XB*-binding sites, or activation of a *Tn*, automatically implies *XB* formation in the environment of that *Tn*. After formation, the *XB* exerts a longitudinal force *F*_*XB*_ on the thin filament which is guided as tension along the thin filament to the Z-disk (Fig 3). Thus, toward the Z-disk, the longitudinal tension *S*(*x*) in the thin filament is increasing by a step *F*_*XB*_ at each location where a *XB* has formed. Variable *x* represents the distance from the beginning of the single overlap region moving toward the Z-disk (Fig 3). The maximum value (*x*_*max*_) of *x* represents the location on the thin filament where the single overlap region ends. The single overlap length depends on *SL* and the assumed filament lengths as shown in S1 Appendix.

Looking from the middle of the sarcomere towards the Z-disk, for tension *S*(*x*_*j*_) just distal to *XB*_*j*_ at position *x*_*j*_ it holds:

$$S\left(x_{j}\right)=\;\sum_{i=1}^{i=j}F_{XB,\;i}\;\;with\;1\leq j\leq j_{max}$$ (2)

The symbol *j*_*max*_ indicates the number of *XB*’s attached to the thin filament in the single overlap region. Eq (2) is graphically elucidated in Fig 3. Tension *S*(*x*) increases with each attached *XB* until distance *x* exceeds the end of the single overlap region where no *XB*'s can be formed. The total tension of the thin filament equals the tension acting on the Z-disk from that thin filament.

### Implementation of the MechChem model

The following assumptions are key to the MechChem model implementation presented in this section:

- *Ca*^*2+*^ binding to *Tn* moves *Tm* azimuthally on the thin filament, unblocking the *XB*-binding sites on nearby actin monomers.
- Unblocking of binding sites implies *XB* binding and force development.
- *XB*’s can form solely in the single overlap region.
- The total tension in the thin filament at the Z-disk is the sum of the individual *XB* force acting on the thin filament.
- Tension hinders the deactivation of the thin filament. The energy required for *Tn* to release *Ca*^*2+*^ increases linearly with the mechanical tension in the thin filament.
- Binding of *Ca*^*2+*^ to an in-register *Tn* pair (*Tn*_*2*_) is intrinsically cooperative, characterized by the base cooperativity coefficient *n* (according to [20]: n = 3).
- When simulating isometric tension development, all *XB*’s generate equal force.

Referring to Eq 2, the principle of the model is explained by the existence of discrete locations where *XB*’s can form. In living muscle at any given time during contraction, there are countless *XB*’s. Thus, we decided to represent muscular force generation by the average of many discrete states, allowing us to describe tensions as continuous functions of distance *x* along the representative thin filament. Focusing on the static conditions only, the involved chemical reactions are considered in equilibrium. According to Eq 1, we consider binding of *Ca*^*2+*^ to *Tn* as a chemical binding to an in-register *Tn* pair, named *Tn*_*2*_.

Equilibrium concentrations of *Ca*^*2+*^, *Tn*_*2*_, and *Tn*_*2*_*Ca*_*n*_ are related by:

$$K_{TnCa}{}^{n}=\;\frac{\left[Tn_{2}\right]{\left[Ca^{2+}\right]}^{n}}{\left[Tn_{2}Ca_{n}\right]}$$ (3)

The symbol *K*_*TnCa*_ represents the equilibrium constant, and *n* is the base cooperativity coefficient representing the intrinsic cooperativity of *Ca*^*2+*^ binding to *Tn*_2_. We assume that when *Ca*^*2+*^ binds to the *Tn*_*2*_, the nearby *XB*-binding sites are unblocked. For the proportion of activated *Tn*’s, *P*(*x*), at position *x* along the thin filament, it holds

$$P\left(x\right)=\;\frac{\left[Tn_{2}Ca_{n}\right]\left(x\right)}{\left[Tn_{2}]\left(x\right)+[Tn_{2}Ca_{n}\right]\left(x\right)}$$ (4)

Solving Eq (3) for [*Tn*_*2*_*Ca*_*n*_] and substituting the result into Eq (4) renders an expression for *P*(*x*) as a function of [*Ca*^*2+*^] and *K*_*TnCa*_ (Eq 5).

$$P\left(x\right)=\;\frac{1}{1+\;{\left(\frac{K_{TnCa}\left(x\right)}{\left[Ca^{2+}\right]}\right)}^{n}}$$ (5)

Because in the MechChem model binding of *Ca*^*2+*^ to *Tn*_*2*_ is assumed to depend on mechanical tension in the thin filament, the equilibrium constant *K*_*TnCa*_ is considered to depend on the position *x* along the thin filament. The energy required to detach *Ca*^*2+*^ from the *Tn* is assumed to increase linearly with the tension *S*(*x*) in the thin filament. Thus, the equilibrium constant is multiplied by the exponential of the product of tension *S(x*) and a constant *C*_*s*_, representing the effect of the added affinity for *Ca*^*2+*^ by an increase of tension. The latter relation is based on the general physical principle that in equilibrium the ratio of state concentrations is proportional to the exponential of a constant multiplied by the energy difference between both states. Analogously, the ratio of ion concentrations on both sides of a membrane depends on the voltage difference across the membrane. So, we express the dependence of the equilibrium concentration constant, *K*_*TnCa*_ on tension *S*(*x*) by:

$$K_{TnCa}\left(x\right)=\;K_{TnCa0}e^{-C_{s}S\left(x\right)}$$ (6)

The symbol *K*_*TnCa0*_ represents the equilibrium constant in the absence of tension in the thin filament. The physical dimension of constant *C*_*s*_ is the inverse of tension. Replacing *K*_*TnCa*_(*x*) in Eq (5) with Eq (6) yields the tension-dependent expression for *P*(*x*) in Eq (7).

$$P\left(x\right)=\;\frac{1}{1+\;e^{n\left(-C_{s}S\left(x\right)-ln\left(\frac{\left[Ca^{2+}\right]}{K_{TnCa0}}\right)\right)}}$$ (7)

We assume that the density of attached *XB*’s is proportional to *P*(*x*). Assuming that all attached *XB*’s exert the same force during steady state isometric contraction, we find that the *XB*–induced force density *f*_XB_(*x*) acting at location *x* is proportional to *P*(*x*) (Eq 8).

$$f_{XB}\left(x\right)=\;C_{f}P\left(x\right)$$ (8)

Constant *C*_*f*_ represents force density with full *Tn* activation, having the physical dimension of force per unit length along the thin filament. After elimination of *P*(x) by substitution of Eq 7 into Eq 8 and using the property that the derivative d*S*(*x*)/d*x* of tension with respect to *x* equals force density *f*_XB_(*x*), we find the following differential equation for *S*(*x*) with boundary condition *S*(0) = 0:

$$\frac{dS\left(x\right)}{dx}=\;\frac{C_{f}}{1+\;e^{n\left(-C_{s}S\left(x\right)-ln\left(\frac{\left[Ca^{2+}\right]}{K_{TnCa0}}\right)\right)}}$$ (9)

Assuming *n* = 3 according to the findings by Sun et al. [20] for the intrinsic thin filament cooperativity, Eq (9) contains 3 independent parameters, i.e., *C*_*f*_, *C*_*s*_ and *K*_*TnCa0*_.

We have fitted the Hill-type model with the experimental data of Dobesh et al. [18] for comparison, expressing tension *S*_*H*_ as a function of [*Ca*^*2+*^]. The three parameters *EC*_*50*_, *n*_*H*_, and *S*_*max*_, represent the [*Ca*^*2+*^] at the 50% level of maximum tension, the Hill coefficient, and maximum tension, respectively. Thus we used

$$S_{H}=\;S_{max}\frac{{\left[Ca^{2+}\right]}^{n_{H}}}{EC_{50}^{n_{H}}+{\left[Ca^{2+}\right]}^{n_{H}}}$$ (10)

### Simulation protocol

Under normal conditions, cardiac muscle cells are enclosed by membranes that regulate the influx of *Ca*^*2+*^ ions from the extracellular space through channels and pumps [28]. Submersing the muscle in detergent causes perforations in the cellular membranes, a procedure known as muscle skinning. Due to these perforations, the membrane channels and pumps no longer regulate intracellular ion concentrations. Thus, it is assumed that when a skinned muscle cell preparation is submerged in a solution containing ions, the intracellular ion concentrations are equal to that of the immersing solution. Thus, the intracellular [*Ca*^*2+*^] can be manipulated, and the muscle will contract and generate tension in response.

Data points published by Dobesh and colleagues (Fig 2A of the original article) [18] provided the experimentally measured tension values (*S*_*exp*_) at various *SL*’s and [*Ca*^*2+*^]’s. We tested our model at a range of [*Ca*^*2+*^]’s between 0.001 and 10 μM for five *SL*’s ranging from 1.85 to 2.25 μm. Single overlap length *x*_*max*_ increases with SL according to the formulation of Rice et al [22], the equations of which are presented in the S1 Appendix (Eq A1-A3). The parameters *C*_*s*_, *C*_*f*_ and *K*_*CaTn*0_ were varied so that the sum of the squared differences between the experimental data by Dobesh and the solution of the differential equation (Eq 9) was minimal. The minimization was performed on each individual curve. The Hill-type model with parameters *EC*_*50*_, *S*_*max*_, and *n*_*H*_ was fitted to the same experimental data. For the MechChem and Hill-type models, the residual errors were assessed to find systematic differences between model and measurement. Additionally, the root mean squared error (*RMSE*) was calculated for all individual curves (Eq 11). *S*_*model*_ and *S*_*Dobesh*_ represent the tension generated in the model and the tension measured experimentally by Dobesh et al., respectively. Because the tension values reported by Dobesh et al. referred solely to the active tension generated, the results of the MechChem model are also reported as active tension. Additionally, we calculate the tension solely in the thin filament. Hence, the passive tension component contributed by the extracellular matrix or titin is viewed as a separate component that would be additional to the calculated active tension. The number of points is represented by *j*.

$$RMSE=\;\sqrt{\frac{\sum_{i=1}^{j}{\left(S_{model}-S_{Dobesh}\right)}^{2}}{j}}$$ (11)

The *RMSE* compares the results of our model to the data from the experiments of Dobesh et al. [18].

In the MechChem model, tension was obtained by solving Eq 9 numerically in Matlab (MathWorks, Natick, MA) with the ode23 solver.

## Results

**Fig 4:**
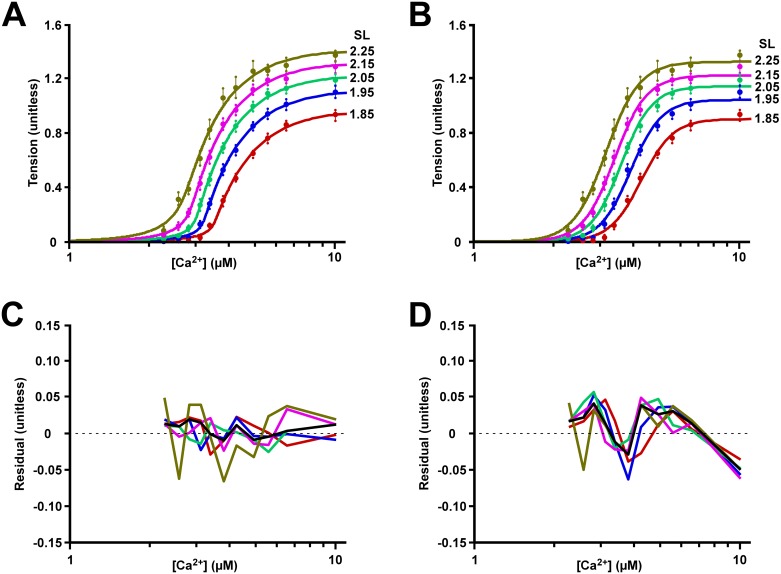
Static [*Ca*^*2+*^]-tension relationship in skinned muscle. (A). The MechChem model generated [*Ca*^*2+*^]-tension relationship with all parameters fitted per curve. The dots on the curve are plotted from the data presented in Table 2 and Fig 2A of Dobesh et al. 2002 [18]. (B) Hill model generated [*Ca*^*2+*^]-tension curves. The experimental data points from Fig 4A were superimposed on the model generated curves. The error between the experimental data and both the (C) MechChem model generated [*Ca*^*2+*^]-tension curves and (D) the Hill model generated [*Ca*^*2+*^]-tension curves is shown.

The experimental data of Dobesh and colleagues in skinned cardiac muscle (Table 2 and Fig 2A of the original article) [18] is compared to the MechChem (Fig 4A) and the Hill (Fig 4B) model-generated [*Ca*^*2+*^]-tension relationships in Fig 4. In Fig 4A best-fit curves are shown, as obtained by the MechChem model after proper adjustment of the three parameters *K*_*TnCa0*_, *C*_*f*_ and *C*_*s*_ per curve. Similarly, in Fig 4B, the curves are best-fit simulations, obtained by the Hill-type model, after proper adjustment of parameters *EC*_*50*_, *n*_*H*_ and *S*_*max*_. The lower panels, 4C and 4D, show the fitting errors per curve as a function of the [*Ca*^*2+*^] for the MechChem and Hill-type model, respectively. The black lines indicate the median value of the error. For the MechChem model, there is no clear common pattern, and the error does not significantly differ from zero. For the Hill-type model, the error values show a clear common pattern, showing a consistent underestimation at the beginning of the upslope and the very last data points and a consistent overestimation at the location where the curves bend towards the maximum value. Thus, in contrast with the MechChem model, the shape of the tension curve, as simulated with the Hill-type model, is clearly different from the measured data. The MechChem curves are asymmetric with a relatively sharp upward bend for low [*Ca*^*2+*^] and a moderate bending toward the saturation level.

In Table 1, the triplets of parameter values for the simulated curves are shown per sarcomere length for both the MechChem model and the fits according to the Hill-type model. The best-fit values we obtained for the Hill-type model were similar to those obtained by Dobesh et al. [18]. The differences we report are likely a result of either a different fitting routine or slight errors when the data were extracted from a published figure. On average, the root mean square error *RSME* for the MechChem model is smaller than that for the Hill-type model. The parameter *C*_*f*_ varied by a maximum of 6.5% at different *SL*’s, whereas *K*_*TnCa0*_ and *C*_*s*_ decreased by 43.2% and 70.6%, respectively. Interestingly, for the MechChem model, parameter values *K*_*TnCa0*_ and *C*_*s*_ exhibit strong interdependency. Our analyses showed that for a change in *K*_*TnCa0*_ by a factor of *a* (*a*>1), *C*_*s*_ changed according to Eq 12 while exhibiting little influence on the resulting [*Ca*^*2+*^]-tension relationship.

$$a*K_{TnCa0}\approx a^{3}*C_{s}$$ (12)

**Table 1:** Parameter values of fit model to experimental data.

Sarcomere length	μm	1.85	1.95	2.05	2.15	2.25
**MechChem n = 3**						
*K*_*TnCa0*_	μM	10.09	9.46	8.63	7.14	5.73
*C*_*f*_	10^6^∙m^-1^S	1.85	1.95	1.97	1.96	1.95
*C*_*s*_	S^-1^ ^1)^	9.27	8.66	7.32	4.61	2.73
RMSE	S	0.017	0.013	0.012	0.017	0.040
**MechChem n = 3 with fixed: *C***_***f***_ **= 1.93∙10**^**6**^ **m**^**-1**^**S, *C***_***s***_ **= 6.5 S**^**-1**^						
*K*_*TnCa0*_	μM	9.26	8.56	8.18	7.91	7.48
RMSE	S	0.024	0.018	0.019	0.021	0.053
**Hill-type**						
*Ca*_*50*_	μM	4.28	3.9	3.6	3.4	3.16
*n*_*H*_	-	7.3	7.5	7.5	7.2	6.9
*S*_*Max*_	S	0.90	1.04	1.14	1.22	1.32
RMSE	S	0.028	0.037	0.033	0.032	0.033

^1)^ S = unit of sarcomere tension, used by Dobesh et al.

**Fig 5:**
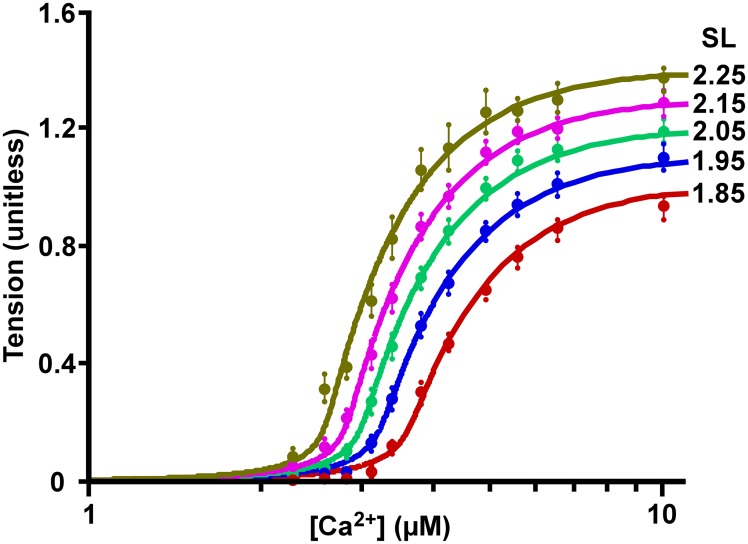
MechChem generated [*Ca*^*2+*^]-tension relationship in skinned muscle. The MechChem model generated [*Ca*^*2+*^]-tension relationship with *K*_*TnCa0*_ changing with *SL*, while setting Cf = 1.93∙10^6^ m^-1^S and Cs = 6.5 S^-1^. The unit S refers to the unit of tension as shown by Dobesh et al. [18]. The dots on the curve are plotted from the data presented in Table 2 and Fig 2A of Dobesh et al. 2002 [18].

According to the abovementioned interdependency, it is possible that either *K*_*TnCa0*_ or *C*_*s*_ changes with *SL*. We assume that *K*_*TnCa0*_ is the parameter that changes with *SL* because the necessary increase in *K*_*TnCa0*_ is much less than that in *C*_*s*_. Additionally, a large body of research has shown that the sensitivity of *Tn* to bind *Ca*^*2+*^ changes with *SL* [18, 29, 30]. Therefore, we performed a best fit by varying *K*_*TnCa0*_ while keeping *C*_*f*_ and *C*_*s*_ fixed at their average values. The resulting fit is shown in Fig 5. As shown in Table 1, the fit is nearly as good as the fit by variation of all three parameters. For the Hill-type model, all parameters vary with sarcomere length, albeit that cooperativity only slightly diminishes with sarcomere length.

In the results, obtained with the MechChem model, cooperativity is apparent from the steep upslope of the [*Ca*^*2+*^]-tension relationship (Fig 4A), i.e. the sharp upward bend in the [*Ca*^*2+*^]-tension relationship that begins at [*Ca*^*2+*^] of about 2.5 μM. With increasing *SL*, there is an increase in both maximum tension development and slope in both the model-generated and experimental [*Ca*^*2+*^]-tension relationships. Furthermore, the [*Ca*^*2+*^] required for half maximum tension development (*EC*_*50*_) decreases with longer *SL*’s indicating the increasing sensitivity of tension development in the thin filament to *Ca*^*2+*^. The experiments of Dobesh et al. [18] showed a decrease in *EC*_*50*_ from 4.28 μM to 3.16 μM with *SL* increasing from 1.85 μm to 2.25 μm (as determined by the Hill fit in our analyses) (Fig 4B, Table 1). In the MechChem simulations covering the same SL range, *EC*_*50*_ decreased from 4.18 μM to 3.17 μM when fitting the model to the [*Ca*^*2+*^]-tension curves per SL (Fig 4A).

**Fig 6:**
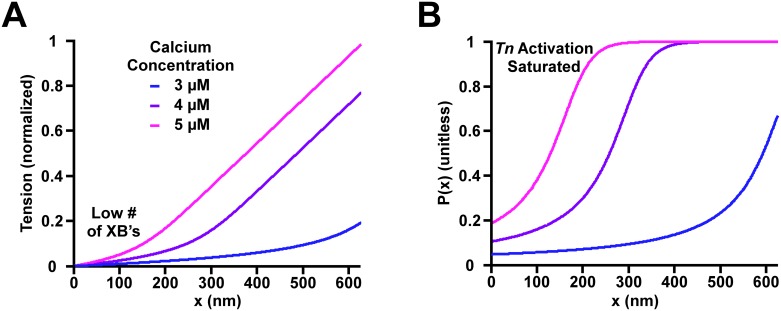
Tension and the proportion of activated troponin complexes (*Tn*) *P*(*x*) in the thin filament along the single overlap region. A sarcomere with length 2.05 μm was modeled with constant calcium concentrations ([*Ca*]). The blue, purple, and magenta lines represent a [*Ca*] of 3, 4, and 5 μM, respectively. (A) The plot shows the non-linear cumulative tension developed at position *x* along the single overlap region. (B) *P*(*x*) is displayed. The plateaus reached at [*Ca*]’s of 4 and 5 μM represent the full activation of *Tn*’s, i.e. all binding sites on that section of the thin filament are exposed for *XB* binding.

Fig 6A and 6B show the MechChem simulation of tension and degree of activation along the thin filament in the single overlap zone, respectively, at different [*Ca*^*2+*^]’s (3, 4, 5 μM with SL = 2.05 μM). These values were chosen to show the results in the middle range of the experimental conditions. The MechChem model predicts that the density of bound *XB*’s increases with the position *x* along the thin filament. The plateaus shown in Fig 6B represent the full activation of *Tn*’s, i.e. all binding sites on that section of the thin filament are exposed for *XB* binding.

## Discussion

The methods presented here provide a novel view of a mechanochemical mechanism of cooperativity in cardiac sarcomeres. The intrinsic cooperativity of *Ca*^2+^ binding to an in-register pair of *Tn*’s, i.e., cooperativity in the absence of mechanical tension, is characterized by a base cooperativity coefficient *n* = 3 according to experimental data reported by Sun et al. [20]. In skinned muscle experiments of Dobesh et al. [18], cooperativity is considerably higher, as characterized by a Hill coefficient of 7. We propose that the latter boost occurs by a mechanism where mechanical tension in the thin filament strengthens the chemical binding of *Ca*^*2+*^ to *Tn*. With each *XB* bound, the tension in the thin filament increases toward the Z-disk. This tension increments the energy required to deactivate a *Tn*, thus increasing the affinity of *Ca*^2+^ for *Tn* towards the Z-disk. The model-simulated [*Ca*^*2+*^]-tension relation appeared in good agreement (Table 1) with the experimental data from Dobesh et al. [18], thus supporting the hypothesis that an important contribution to cooperativity in cardiac muscle is delivered by the mechanical tension in the thin filament acting over a long range, i.e. the entirety of the thin filament.

### Comparing measured [Ca^2+^]-tension relation with MechChem model simulations

Considering the MechChem simulation with parameters *C*_*f*_ and *C*_*s*_ fixed and *K*_*TnCa0*_ depending on SL (Fig 5, Table 1), the model-generated [*Ca*^*2+*^]-tension curves resembled the experimental data of Dobesh et al. [18]. Typically, the log[*Ca*^*2+*^]- tension curve is S-shaped from zero tension at low [*Ca*^*2+*^] to a saturated value at high [*Ca*^*2+*^]. The steepness of the slopes in the experimental data of Dobesh et al. [18] indicates cooperativity with a Hill coefficient around 7. At low [*Ca*^*2+*^], the [*Ca*^*2+*^]- tension bends up sharply, giving rise to the steepest part of the curve, indicating a high [*Ca*^*2+*^] sensitivity. With maximum [*Ca*^*2+*^] (10 μM) and SL increasing from 1.85 μm to 2.25 μm, the simulated peak tension increased by 49% and the measured value by 47%. Simulations also produced an increase in *Ca*^*2+*^ sensitivity, shown by a decrease of *K*_*CaTn0*_ from 16.6 down to 11.8 μM, implying a leftward shift of the [*Ca*^*2+*^]-tension curves with increasing *SL*. The shape of the MechChem curves (Figs 4A and 5) covers the experimental data better than the sigmoid Hill-curves (Fig 4B), as shown by the residuals in Fig 4C and 4D, especially for the mid and high [*Ca*^*2+*^]. It should be noted that for low [*Ca*^*2+*^] the residuals of the MechChem curves are somewhat larger, probably because of the steep gradient of that part of the curve. The discrepancy between the results of the MechChem model at low [*Ca*^*2+*^] and the experimental results may be further explained by assuming some dispersion in sarcomere length and in the length of the thin and thick filaments, causing the sharp uprising bend to be smeared out, thus moderating the slope in that part of the curve.

### Proposed mechanism of cooperativity in the MechChem model

Cooperativity is assumed to have two components. The first is the intrinsic cooperativity related to binding of *Ca*^*2+*^ to *Tn* in absence of mechanical tension. The related intrinsic cooperativity coefficient is set to 3 according to the findings by Sun et al. [20]. Secondly, the mechanochemical component boosts the cooperativity from the intrinsic coefficient of 3 up to the experimentally measured value of about 7.

Sun et al. [20] inhibited tension development in trabeculae with blebbistatin and found that in the absence of tension the Ca-dependent activation of *Tn*’s along the thin filament remained cooperative with a Hill coefficient *n*_*H*_ = 3. The Hill coefficient indicates the minimum number of binding sites that cooperate. Each *Tn* contains 3 binding sites for *Ca*^*2+*^ [20], but they do not all have the same affinity and are not likely to fully cooperate. Galińska-Rakoczy et al. [17] showed that the C-terminal section of *TnI* touches the *Tm* on the adjacent actin strand, thus coupling both *Tn*’s, forming an in-register pair *Tn*_2_. Consequently, for each *Tn*_*2*_, there are 6 binding sites available for inherent cooperation. Apparently, cooperativity is partial, resulting in *n* ≈3.

By introduction of the MechChem model of tension-driven cooperativity, we propose that mechanical tension in the thin filament results in the strengthening of the binding between *Ca*^*2+*^ and *Tn*, thereby hindering deactivation, implying that tension lowers the energy associated with *Ca*^*2+*^ being bound to *Tn*. Combining intrinsic cooperativity with the mechanochemical mechanism results in the MechChem model, which results display a striking resemblance to the experimentally measured tension as a function of [*Ca*^*2+*^] in skinned muscle preparations (Figs 4 and 5). Many correlations between change of *Ca*^*2+*^ binding properties and mechanical events have been reported.

Rieck et al. [31] showed that even in the absence of tension in the thin filament, the formation of strong *XB*’s stabilized the open conformation of the *Tn*. Since *XB*’s can only form if a nearby *Tn* is in the unblocked state, that *Tn* may help to unblock the in-register *Tn*, thus enhancing the formation of additional *XB*’s attached to the paired actin strand. Furthermore, Isambert et al. [32] showed that the rigidity of the thin filament decreased when *Tm* was in the unblocked state as compared to being in the blocked state. This finding shows that there is a mechanical coupling between the conformational changes of *Tn* and elastic properties of the thin filament. More recently, Desai et al. [33] directly showed that myosin binding was necessary for complete activation of the thin filament upon partial activation due to calcium binding.

The MechChem simulated best-fit [*Ca*^*2+*^]-tension curve was determined for intrinsic cooperativity coefficients *n* = 1,2,3 and shown in the supplementary material (S1 Fig). The fit is excellent when *n* = 3, while for *n* = 1 or 2, the curves are not as steep as in the physiological situation. In the experiments by Sun et al., development of mechanical tension was shown not to have an effect on cooperativity, i.e. the Hill coefficient remained as low as 3. As they mentioned in their article [20], they could not exclude the possibility that the applied fluorescent probes attached to the various structures of the *Tn* complex may change the properties of *Tn* to some degree. We think that these probes may inhibit the mechanochemical enhancement of *Ca*^*2+*^ affinity by tension.

### Cooperativity in the MechChem model vs. the Hill model

The [*Ca*^*2+*^]-tension relation in skinned muscle has generally been characterized by a modified Hill curve that takes on a symmetric s-shape [34]. The [*Ca*^*2+*^]-tension relation simulated by our model is asymmetric (Fig 4A and 4C); the initial steep rise in tension generated with additional *Ca*^*2+*^ decreases closer to saturation. Whereas Dobesh et al. fitted symmetric curves, they admitted that the Hill curves consistently overestimated the tension developed in the sarcomeres as the curves rounded toward saturation (Fig 4B and 4D). In an attempt to account for the asymmetry, Dobesh et al. [18] proposed that the experimental data be fit to 2 Hill coefficients that meet at *EC*_*50*_ for each curve. In the Mech Chem model, the [*Ca*^*2+*^]-tension relation is already asymmetric, so the introduction of an additional parameter is not necessary to reshape the curve.

Both the Hill-type model and the MechChem model require 3 parameters per curve. By fitting the models to the experimental data for each sarcomere length separately, in the Hill-type model all 3 parameters appeared to depend on SL, albeit that dependency of the Hill coefficient *n*_*H*_ appeared weak (Table 1). In the MechChem model, parameters *C*_*f*_ and *C*_*s*_ did not depend clearly on SL, thus hinting us to keep these parameter values fixed, while only reestimating the equilibrium constant *K*_*CaTn0*_, expressing Ca^2+^ affinity in absence of mechanical tension. The resulting fits were nearly as good (Fig 5), suggesting that Ca^2+^ affinity apparently depends somehow on SL, while the other parameters were general to all SL’s.

Our model comprises two mechanisms of cooperativity that generate the [*Ca*^*2+*^]-tension relationship, i.e. tension in *Tm* strengthening the bond between *Ca*^*2+*^ and *Tn* and intrinsic cooperative activation of in-register pairs of *Tn*. The Hill function is based on common chemical equilibrium and provides the basis for many current models of *Ca*^*2+*^-*Tn* binding. For example, Rice et al. [22] utilize a modified Hill function to model the transition between the active and inactive states of *Tn*. The peak intracellular [*Ca*^*2+*^] reaches 1.45 μM in the Rice model, a concentration that the Hill function mimics well. The Hill model does not provide a physical explanation for the mechanism of cooperativity in cardiac muscle, but instead utilizes a coefficient that characterizes the steepness of the [*Ca*^*2+*^]-tension relation. Additionally, the Hill model was initially developed to understand the cooperative binding of oxygen to hemoglobin [34], where it makes physical sense that a first chemical binding of oxygen on a hemoglobin molecule will facilitate subsequent bindings because oxygen-binding sites on hemoglobin are separated by only 2.5 to 3.5 nm [35]. To reach the high physiologic level of cooperativity, several *Tn*’s must interact. Because *Tn*’s are separated by about 35 nm along a single actin strand within the thin filament, it is physically difficult to explain that a chemical binding can influence the binding of a different molecule that far away by conventional chemical principles.

It has been shown that the sensitivity of *Tn* to bind *Ca*^*2+*^ changes with *SL* [18, 29], a characteristic implemented in the MechChem model. There is currently no clear consensus regarding the mechanism behind length dependent *Ca*^*2+*^ sensitivity in cardiac muscle (for review, [36]). One proposed mechanism is that the lattice spacing decreases with longer *SL*, moving myosin heads closer to the thin filament and rendering the thin filament more sensitive to *Ca*^*2+*^ by enhancing binding [37]. However, the lattice spacing hypothesis has been questioned after it has been shown that muscle length does not necessarily correlate with myofilament spacing [30]. It has also been proposed that phosphorylation of sites on *TnI* by protein kinase A and protein kinase C alters the *Ca*^*2+*^ sensitivity and could prove significant in the regulation of length dependent activation [14]. Conversely, Lee et al. have shown that the increase of passive tension in titin leads to increased *Ca*^*2+*^ sensitivity [38]. It is possible that the change in *K*_*TnCa0*_ in the MechChem model is due to a combination of the abovementioned mechanisms.

### The MechChem model vs. nearest neighbor cooperativity models

As already indicated in the introduction, various models have been developed on the basis of nearest neighbor cooperativity, based on *RU*-*RU*, *XB*-*XB* or *XB*-*RU* interactions. These models generally result in Hill-type [*Ca*^*2+*^]-tension relations that fit accurately to experimental data. However, the curves are slightly, but systematically different from the MechChem curves (Fig 4). It is not clear yet if these differences are sufficiently strong to make a choice between the two model types. We predict a clear difference to be expected, yet we do not currently have the means to test the model. In a tension bearing sarcomere, the MechChem model predicts that the hindrance to *Tn* deactivation imposed by high tension in the thin filament causes a higher concentration of activated *Tn*’s, bound *Ca*^*2+*^, and *XB*’s toward the Z-disk end of the single overlap region where tension is highest (Fig 5B). With the nearest neighbor hypothesis, no preference is to be expected on the location of activated *Tn*’s. However, although not explicitly noted, the MechChem model does take into account some of the nearest neighbor cooperativity mechanisms. The development of tension in the thin filament begets more tension development. Thus, *XB*’s recruit more *XB*’s. Additionally, the tension in the thin filament at position *x* strongly determines whether the *Tn* is active at point *x* (*XB*-*RU* cooperativity). *RU*-*RU* cooperativity is accounted for in the MechChem model through the intrinsic cooperativity coefficient *n*.

### Comparison with long range cooperativity hypotheses

Most of the models previously discussed have included only local cooperativity mechanisms, yet there are also models and hypotheses that incorporate cooperative mechanisms acting along the entirety of the thin filament. Brandt and colleagues [39] proposed that the mutual overlap of *Tm* molecules under *Tn* causes a simultaneous unblocking or blocking of all *Tm* molecules along the filament. Conversely, the “cooperative realignment of binding sites”, a model developed by Daniel and colleagues [26], ignores the effects of *Tm* but looks instead at the possible impact of strain on the thin filament. Hence, this model can predict cooperativity in tension development but not in activation. Still others attribute the cooperative effect to the constant volume property of the myofiber matrix. The stretch of sarcomeres causes the thick and thin filaments to squeeze closer together increasing the probability of *XB* binding [24]. Like the cooperative realignment of binding sites, the lattice spacing hypothesis can account for cooperative tension generation but not activation.

The model designed by Land & Niederer [40] represented the entire thin filament, a compilation of 26 *RU*’s. They hypothesized that the state (position) of the *Tm* molecule in each *RU* has a corresponding free energy determined by the state of neighboring *XB*’s and *RU*’s. The energy term is used to compute the probability of a *RU* being blocked or unblocked. While the simulated results are consistent with available experimental data and insight can be gained through the Land & Niederer model, it is composed of a system of 750 ordinary differential equations. The tension-driven cooperativity model we present consists of a single ordinary differential equation and three key parameters, so we present a highly simplified model that still captures behaviors shown in available experimental data. Our model, like the Land & Niederer [40] model, includes an energy term in the computations of state *P*. It is a mechanical energy term related to the tension within the thin filament that increases along the thin filament from the start of the single overlap region towards the Z-disk. Our model is different from others because the mechanics of the thin filament (*S*(*x*)) directly impact the related chemistry (*Ca*^*2+*^ binding to *Tn*) whereas most of the other models view the mechanics and chemistry separately.

Izakov et al. have hypothesized that the number of strongly bound *XB*’s along the thin filament affects the binding affinity of *Ca*^*2+*^ to *Tn* and have implemented this idea in a computational model [41]. Landesberg and Sideman proposed a similar model, but it was a loosely coupled model meaning that *Ca*^*2+*^ was not required to remain bound for the *Tn* to remain active, but bound *XB*’s were adequate to do so [42]. Our model differs because we propose that the tension in the thin filament contributes to an increment energy required to unbind *Ca*^*2+*^ from *Tn*. This energy increases along the thin filament toward the Z-disk as tension increases. In the models discussed above, the global affinity for *Ca*^*2+*^ to bind to *Tn* will change based on the number of strong *XB*’s. Within our model, however, the affinities increase with tension in the thin filament, being explained by a linear increase of binding energy with tension in the thin filament.

### Mechanism of relaxation as calcium concentration decreases

Due to the interaction between mechanics and chemistry within the model, neither mechanics nor chemistry fully account for the activation or subsequent deactivation of the thin filament. While we propose that high tension in the thin filament hinders deactivation, a decrease in intracellular [*Ca*^*2+*^] will trigger relaxation. Due to the buildup of tension within the thin filament as shown in Fig 3, there is always a loose end closer to the mid-line in which little tension is developed. The hindrance to deactivation imposed by high tension does not exist in these areas, thus promoting the deactivation of the *Tn*’s closest to the mid-line first. We propose that the areas of highest tension (closest to the z-disk) are the latest to deactivate.

### Heterogeneity of cross-bridge density

Our model has provided a potentially experimentally testable hypothesis. Model results suggest directionality in the dispersion of *XB*’s along the thin filament with a higher concentration of *XB*’s closer to the Z-disk. Desai and colleagues [33] were recently able to fluorescently label single myosin heads and observe single bindings to the thin filament. They observed that although myosin binding activated the *RU*, there was no directionality in the binding of *XB*’s. However, tension was not developed in this model because the myosin heads were not tethered to the thick filament. Additional experimental evidence is needed to test the hypothesis of *XB* dispersion in the loaded thin filament. The difficulty lies in developing an experimental approach that enables viewing of each individual *XB* binding in a skinned muscle under tension.

### Model limitations

The model presented here assumes that when an area on the thin filament becomes unblocked, *XB*’s are automatically formed. Thus, the fraction of unblocking is proportional to the *XB*- force developed along the strand. The assumption that *XB*’s form automatically when *RU*’s are activated is not physiologically accurate. We expect this to be corrected upon the explicit incorporation of the different steps of the *XB* cycle. However, the experimentally measured tension in skinned muscle preparations is obtained after the steady state has been reached. Hence, the experimental data is time-independent.

The current model results are limited to the [*Ca*^*2+*^]- tension relationship. The introduction of dynamics such as the *XB* cycle requires additional assumptions to be made and additional unknown parameters added. However, the next logical step for the model is the implementation of the *XB* cycle that will be studied in the isometric twitch. The MechChem model is a mean field approximation, so it is highly simplified. Specific spatial details such as individual binding sites are not accounted for, but the simplification reduces computational cost considerably.

### Conclusions

A novel mechanochemical model of tension generation by the sarcomere has been developed based on long range cooperativity imposed by mechanical tension in the thin filament and intrinsic cooperativity resulting from the interaction between the calcium-binding sites on the in-register troponin complexes. Simulated [*Ca*^*2+*^]-tension curves resembled those obtained in steady state isometric muscle experiments. Thus, our results support the hypothesis that high tension in the thin filament impedes deactivation by increasing the energy required to detach calcium from the troponin complex. Furthermore, we found that the tension in the thin filament was relatively low toward the beginning of the single overlap region close to the mid-line of the sarcomere but increased steeply in the overlap region closer to the Z-disk. Model simulations suggest that the concentration of calcium bindings to the troponin complexes and active *XB*’s are low at the free end of the thin filament and saturated closer to the Z-disk. Future experimental studies are needed to test the latter property, indicating the validity of our hypothesis on the cooperative effect of tension in the thin filament on force generation by the cardiac sarcomere.

## Supporting Information

S1 Fig [Ca^*2+*^]-Tension relationship alterations in intrinsic cooperativity. The cooperativity constant *n* is altered between values of 1 (blue), 2 (green), and 3 (magenta) at *SL* = 2.05 μm. The green dots on the plot are the experimental data of Dobesh et al, and the best fit of the model to the experimental data of Dobesh et al. was found for each *n* [1, 2, 3]. *C*_*s*_ = [134.90, 24.07, 7.32] (S^-1^) *C*_*f*_ = [0.00293, 0.00220, 0.00197] (nm^-1^S) *K*_*TnCa0*_ = [618.97, 22.14, 8.63] (μM).
(TIF)

S1 Appendix Calculation of single and double overlap lengths in a sarcomere. (DOCX)

S1 Model Code Zip file containing the Matlab program representing the MechChem model. (ZIP)
