# Supplementary figures and images for: Mechano-chemical Interactions in Cardiac Sarcomere Contraction: A Computational Modeling Study
Source: PLoS Comput Biol. 2016 Oct 7;12(10):e1005126. doi: 10.1371/journal.pcbi.1005126 (PMC5055322; doi:10.1371/journal.pcbi.1005126)

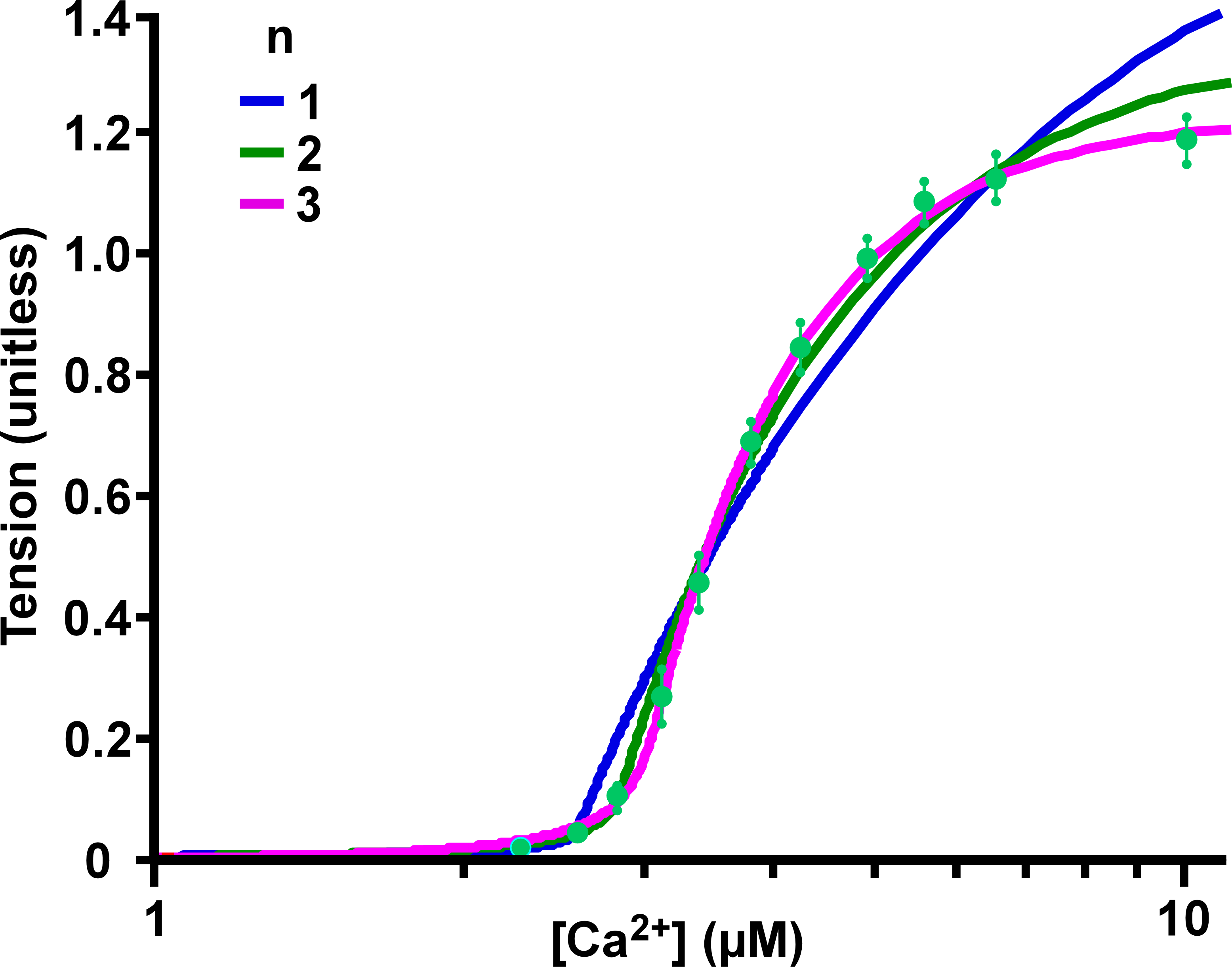

Supplement: S1 Fig — The cooperativity constant n is altered between values of 1 (blue), 2 (green), and 3 (magenta) at SL = 2.05 μm. The green dots on the plot are the experimental data of Dobesh et al, and the best fit of the model to the experimental data of Dobesh et al. was found for each n [1, 2, 3]. Cs = [134.90, 24.07, 7.32] (S-1) Cf = [0.00293, 0.00220, 0.00197] (nm-1S) KTnCa0 = [618.97, 22.14, 8.63] (μM). (TIF) [file pcbi.1005126.s001.tif]
