# Supplementary material for: Mechano-chemical Interactions in Cardiac Sarcomere Contraction: A Computational Modeling Study
Source: PLoS Comput Biol. 2016 Oct 7;12(10):e1005126. doi: 10.1371/journal.pcbi.1005126 (PMC5055322; doi:10.1371/journal.pcbi.1005126)
Supplement: S1 Appendix — (DOCX) [file pcbi.1005126.s002.docx]

## S1 Appendix

## Overlap length

Single overlap length *x_max_* of the thin and thick filaments depends on SL and length of the thin filament *l_thin_*, the myosin filament *l_thick_*, and the bare zone *l_bare_* in the middle of the thick filament according to the formulations of Rice et al [18]:

| $x_{max}=x_{z}-x_{c}$ | (A1) |
| --- | --- |

| $x_{z}=min\left( \frac{l_{thick}}{2}, \frac{l_{sarc}}{2} \right)$ | (A2) |
| --- | --- |

| $x_{c}=max\left( \frac{l_{sarc}}{2}- \left( l_{sarc}-l_{thin} \right), \frac{l_{bare}}{2} \right)$ | (A3) |
| --- | --- |

The values of the independent length components utilized in the model are as follows: *l_thin_* = 1.2 µm, *l_bare_* = 0.1 µm, *l_thick_* = 1.6 µm [9].
